# Grey Matter Correlates of Three Language Tests in Non-demented Older Adults

**DOI:** 10.1371/journal.pone.0080215

**Published:** 2013-11-05

**Authors:** Haobo Zhang, Perminder S. Sachdev, Wei Wen, Nicole A. Kochan, John D. Crawford, Henry Brodaty, Melissa J. Slavin, Simone Reppermund, Kristan Kang, Julian N. Trollor

**Affiliations:** 1 Brain and Ageing Research Program, School of Psychiatry, University of New South Wales, Sydney, New South Wales, Australia; 2 Neuropsychiatric Institute, Prince of Wales Hospital, Randwick, New South Wales, Australia; 3 Academic Department for Old Age Psychiatry, Prince of Wales Hospital, Randwick, New South Wales, Australia; 4 Dementia Collaborative Research Centre, School of Psychiatry, University of New South Wales, Sydney, New South Wales, Australia; 5 Department of Developmental Disability Neuropsychiatry, School of Psychiatry, University of New South Wales, Sydney, New South Wales, Australia; Hangzhou Normal University, China

## Abstract

Language has been extensively investigated by functional neuroimaging studies. However, only a limited number of structural neuroimaging studies have examined the relationship between language performance and brain structure in healthy adults, and the number is even less in older adults. The present study sought to investigate correlations between grey matter volumes and three standardized language tests in late life. The participants were 344 non-demented, community-dwelling adults aged 70-90 years, who were drawn from the population-based Sydney Memory and Ageing Study. The three language tests included the Controlled Oral Word Association Task (COWAT), Category Fluency (CF), and Boston Naming Test (BNT). Correlation analyses between voxel-wise GM volumes and language tests showed distinctive GM correlation patterns for each language test. The GM correlates were located in the right frontal and left temporal lobes for COWAT, in the left frontal and temporal lobes for CF, and in bilateral temporal lobes for BNT. Our findings largely corresponded to the neural substrates of language tasks revealed in fMRI studies, and we also observed a less hemispheric asymmetry in the GM correlates of the language tests. Furthermore, we divided the participants into two age groups (70-79 and 80-90 years old), and then examined the correlations between structural laterality indices and language performance for each group. A trend toward significant difference in the correlations was found between the two age groups, with stronger correlations in the group of 70-79 years old than those in the group of 80-90 years old. This difference might suggest a further decline of language lateralization in different stages of late life.

## Introduction

The neural basis of language has been extensively studied, showing that distinct brain regions play different roles in language processing [1-7]. The temporal lobe functions as a core storage site for phonemic and semantic information [2,5,8,9]; the frontal lobe is involved in executive control and articulatory planning [10-13]; and the parietal lobe is associated with the integration of information regarding various attributes of objects [14,15]. Functional magnetic resonance imaging (fMRI) has been widely used in language studies, as it can directly measure neural activity during language tasks. However, the adaptation of standardized tests to fMRI designs has been constrained. Standardized language tests, specifically verbal fluency and naming tests, have been commonly used by clinical psychologists to assess language function of participants [16]. Both verbal fluency and naming tests require overt speech production that can cause head movements and increase image artefacts, making them unsuitable for fMRI studies [17]. Different from fMRI studies, structural MRI studies investigate the relationships between brain structural measures and language performance, which can provide insights regarding the neuroanatomical basis of standardized language tests [18].

In contrast to abundant functional neuroimaging studies on language, only a small number of structural neuroimaging studies have examined the correlations between grey matter (GM) measures and language performance in healthy adults [18-20]. In participants with a wide age range across young and old adulthoods, language tests have been found to be positively correlated with regional GM measurements. Previous studies have shown that positive GM correlates of phonemic fluency tests were located in the left frontal and temporal lobes [19,20]; and the positive GM correlates of semantic fluency tests were located in the left temporal lobe [19]. The results from structural imaging studies are concordant with the findings of functional neuroimaging studies, as the locations where the GM volumes are positively correlated with language performance are consistent with the brain regions that show functional activations during comparable language tasks.

As ageing is accompanied with brain atrophy and cognitive decline, the structure-language relationship in old adulthood might be different from the relationship in young adulthood. However, only a few studies have examined the correlations between GM measures and language tests in older adults, and their results are rather diverse. One study reported that naming tests were positively correlated with GM volumes in the left temporal lobe [21]. In another two studies, however, no positive correlation was found between GM volumes and verbal fluency and naming tests [22,23]. The inconsistent results may be due to different sample compositions across studies. Several studies have shown that language function remains stable until late old age [24-26]. One recent study demonstrated a late change in the structural cortical network associated with language function [27]. In people of early old age, individual variations on language performance and relevant brain structures may be limited, which reduce the likelihood of a relationship between the two variables [22,23]. The small sample size of studies may also contribute to the inconsistency of results [21,23]. Thus, a further study in a large sample of elderly adults aged 70 and beyond is warranted to better understand the structure-language relationship in late life.

Language has an interesting characteristic as its neural basis often presents an asymmetric pattern, showing a left-hemispheric dominance in the frontal and temporal lobes, termed as language lateralization [28-30]. Most studies on language lateralization employ fMRI to directly compare bilateral neural activities related to a language task. However, structural MRI studies also provide evidence to support language lateralization. Structural-functional correlation analysis has shown that the GM volumes that are positively correlated with verbal fluency tests are mainly located in the left frontal and temporal lobes [19,20]. Moreover, the variation in language lateralization is found to be associated with the degree of structural laterality [31-33]. Evidence also suggests that higher degree of leftward asymmetry in language-related regions is correlated with better language function [34-36]. In older adults, however, the neural basis of language displays a more symmetric pattern, located in the bilateral frontal and temporal lobes [37-39]. Based on substantial evidence provided by fMRI studies, a theory was proposed (the HAROLD model) [40], suggesting that the reduction of hemispheric asymmetry in older adults may reflect the plasticity of the ageing brain to engage extra neural circuits to compensate age-related neural inefficiency [40-42]. A recent fMRI study examined language lateralization in different age groups of healthy adults ranging from 5 to 67 years old, and the results showed a trend of decreasing language lateralization from 25 years old onwards [43]. However, few studies have investigated how language lateralization might change in different stages of late life. 

The present study employed three standardized language tests to evaluate the performance of older adults in verbal fluency and naming ability. The performance on language tests was correlated with GM volumes at the voxel-level across the whole brain, which precludes a priori hypotheses for particular brain regions. Our participants were epidemiologically recruited from community-dwelling non-demented adults. With a big sample size (n=344) and an old age range for the participants (70-90 years), this study allows greater inter-individual variations on language performance and GM volumes, which may increase the likelihood of a relationship between the two variables. We hypothesized that some regional GM correlates of language tests might be located in the right hemisphere, consistent with the reduction of hemispheric asymmetry in old age. Moreover, we divided the whole sample into two age groups (70-79 and 80-90 years old), calculated the correlations between structural laterality indices and language tests for each group, and then compared the correlations between the two age groups. We aimed to explore whether the relationship between structural laterality and language function differs in the two age groups, which may indicate a change of language lateralization in different stages of late life. 

## Methods

### Subjects

The whole sample (n=344) was drawn from Wave 1 of the Sydney Memory and Ageing Study (MAS). The MAS participants (n=1037) were randomly recruited from community-dwelling adults aged 70-90 years [44], with the following exclusion rules: dementia based on DSM-IV criteria [45]; adjusted Mini-Mental State Examination score (MMSE) <24 [46,47]; developmental disability; history of psychosis; multiple sclerosis; motor neuron disease; progressive malignancy; or inadequate English to complete basic assessment. 

For the purposes of this study, additional exclusion criteria were applied to the eligible MAS participants: no MRI scan data (n=495); diagnosed with stroke (n=12), Parkinson's disease (n=8), epilepsy (n=4), severe head injury (unconsciousness > 24 hrs, n=2), brain cancer (n=1), benign meningioma (n=2), brain infection (n=6), transient global amnesia (n=3), or depression (n=60); non-English speaking background (n=78); incomplete data on language tests (n=10); extreme outliers of the language tests scores (> 3 interquartile range below/above 1st/ 3rd quartile) (n=1); or poor MRI scan quality (including MR artifacts, or errors in data saving or converting) (n=11). Of all participants (n=340), 93.3% are right-handers (n=321), 3.2% are left-handers (n=11), and 3.5% are ambidextrous individuals (n=12).

### Ethics Statement

The study was approved by the ethics committee of the University of New South Wales and written informed consent was obtained from each participant.

### Neuropsychological Tests

In the present study, three standardized language tests were administered to all participants by trained psychology graduates. The three language tests were part of a comprehensive battery of neuropsychological tests applied in the Sydney Memory and Ageing study to assess cognitive function of the participants. The Controlled Oral Word Association Task (COWAT) was conducted by asking participants to verbally generate as many words as possible within 60 seconds, beginning with an assigned letter, in this case the letters F, A, and S [48]. The Category Fluency test (CF) required participants to verbally generate as many words from a particular category as possible within 60 seconds, in this study ‘animals’ [49]. The 30 item Boston Naming Test (BNT) consists of 30 picture plates with drawn objects, and required participants to verbally name them [50].

### MRI Acquisition

Structural MRI scans of 184 participants were acquired using a Philips 3T Intera Quasar scanner (Philips Medical Systems, Best, The Netherlands). The remaining 160 participants were scanned on a Philips 3T Achieva Quasar Dual scanner which replaced the original one in 2007 for reasons outside of the investigators’ control. Acquisition parameters for all T1-weighted structural MRI scans were: TR=6.39 ms, TE=2.9 ms, flip angle=8°, matrix size=256x256, FOV=256x256x190, and slice thickness=1 mm with no gap between; yielding 1x1x1 mm3 isotropic voxels. No significant differences on GM, WM, and cerebrospinal fluid volumes were found between the two scanner groups. Moreover, no significant difference in the distribution of two age groups (70-79 and 80-90 years old) was found between the two scanner groups (p=0.24). Nevertheless, a binary variable accounting for each of the scanners was included in the statistical analysis as a covariate to minimize potential scanner effects.

### Image Processing

The procedure for processing T1-weighted MRI scans using the approach of voxel-based morphometry (VBM) had been described previously [51]. In brief, after visual inspection by experienced radiologists the brain scans with structural abnormalities such as brain tumour or severe image artifacts were removed from the study. Secondly we used the hidden Markov random field option in the unified segmentation of the Statistical Parametric Mapping software (SPM5, Wellcome Department of Imaging Neuroscience, London, UK; http://www.fil.ion.ucl.ac.uk/spm) to segment T1 images into different tissues with the most commonly used ICBM152 atlas as the template. Next, the toolbox of Diffeomorphic Anatomical Registration Through and Exponentiated Lie Algebra (DARTEL) [52] in SPM5 was used to generate a series of customized templates and flow fields of GM and white matter (WM) from all T1 images. Each T1 image was then registered to the customized templates to create the modulated warped tissue class image. Then, spatial normalization of GM to the Montreal Neurological Institute (MNI) space was achieved by using an affine transformation to the ICBM152 template. Lastly, the 12-mm full width at half maximum Gaussian kernel smoothing was performed to generate the voxel-based GM volumes for each subject for the subsequent statistical analysis.

### Statistical Analysis

Correlation analyses between voxel-wise GM volumes and language performance were performed in the whole sample (n=344). Using the SPM5 package, the GM volume for each voxel was regressed on the raw score of each test after controlling for age, sex, years of education, total intracranial volume (TIV), scanner, cardiovascular risk score (CVR), and handedness. The calculation of CVR was performed by the MAS research group based on a regression model developed by the researchers of The Framingham Stroke Study [53]. A non-stationary correction toolbox was utilized to overcome the non-stationarity problem in VBM [54,55]. Anatomical locations of the peak voxels were labelled using the SPM Anatomy Toolbox version 1.7 (http://www.fz-juelich.de/inm/inm-1/spm_anatomy_toolbox) [56], and xjView 8 (http://www.alivelearn.net/xjview/). For the voxel-wise GM correlation analyses, the significance threshold in the whole sample was set at a voxel-level inference of p<0.001 (uncorrected) combined with a cluster-level inference of p<0.05 (FWE-corrected). 

A conjunction analysis was performed to locate the common brain areas where GM volumes were positively correlated with all three tests or any two tests in the whole sample, based on the conjunction null method [57]. The positive GM correlates of the three language tests that survived the significance threshold were overlapped with each other, and the common areas were extracted for illustration.

Furthermore, we divided the whole sample into two age groups (aged 70-79 years, n=205; aged 80-90 years, n=139). Based on the results from prior analysis in the whole sample, brain regions where GM volumes were positively correlated with a language test were determined as region-of-interests (ROIs) for that test, and the boundaries of bilateral ROIs were defined using the Automated Anatomical Labelling atlas (AAL) [58]. The structural laterality index (sLI) for each ROI was computed individually with the formula used by previous studies, sLI = (V_left_ - V_right_)/(V_left_ + V_right_) [33]. The values of V_left_ and V_right_ were calculated as the sum of voxel-wise GM volumes within bilateral ROIs. Then the correlation coefficient (r) between sLI and language test was calculated for each age group, after controlling for age, years of education, sex, TIV, scanner, CVR, and handedness (IBM SPSS 20.0, New York). Lastly, to compare the correlation coefficients between two age groups, a Fisher’s z-transformation of the r values was performed and the level of significance was determined [59]. 

## Results

**Figure 1:**
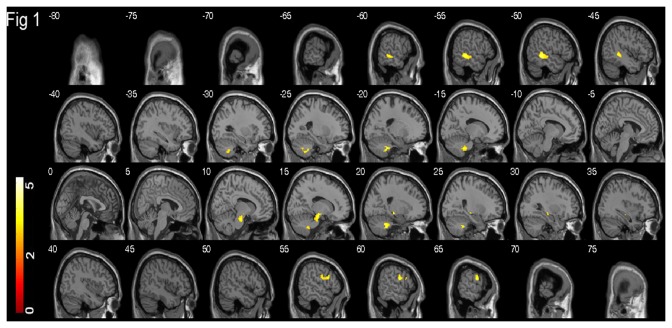
Grey matter correlates of COWAT. Brain regions where voxel-based GM volumes are positively correlated with COWAT in 344 participants aged 70-90 years, are superimposed on the sagittal slices of the brain template. The slices are at 5 mm intervals between and including -80 mm and 75 mm. The colour bar represents the t score ranging from 0 to 5.5; and yellow indicates a higher t score than red.

**Figure 2:**
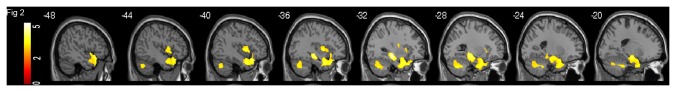
Grey matter correlates of CF. Brain regions where voxel-based GM volumes are positively correlated with CF in 344 participants aged 70-90 years, superimposed on the sagittal slices of the brain template. The slices are at 4 mm intervals between and including -48 mm and -20 mm. The colour bar represents the t score ranging from 0 to 5.5; and yellow indicates a higher t score than red.

**Figure 3:**
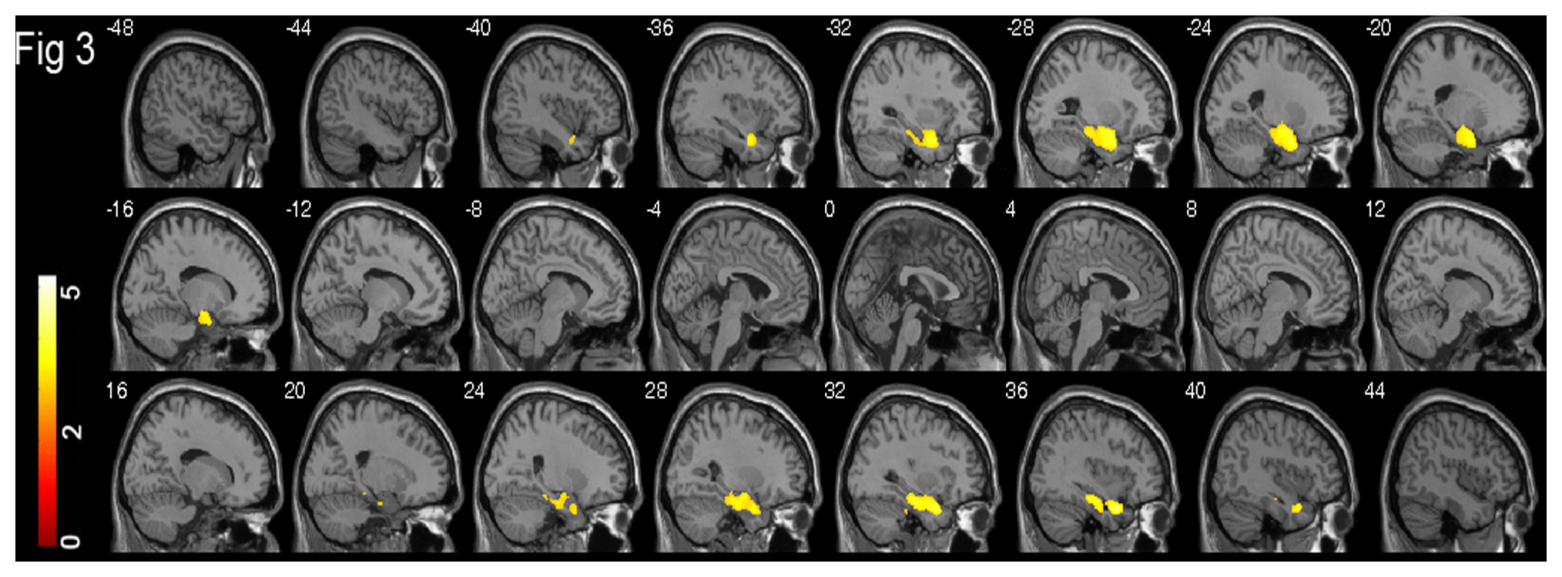
Grey matter correlates of BNT. Brain regions where voxel-based GM volumes are positively correlated with BNT in 344 participants aged 70-90 years, superimposed on the sagittal slices of the brain template. The slices are at 4 mm intervals between and including -48 mm and 44 mm. The colour bar represents the t score ranging from 0 to 5.5; and yellow indicates a higher t score than red.

The demographic characteristics of age, sex and years of education, and the raw scores for each of the three language tests in all participants as well as each age group are shown in Table 1. Correlations between voxel-wise GM volumes and three language test were all positive as no negative correlations were found. The cluster of voxels, where GM volumes were significantly positively correlated with language performance in the whole sample, were superimposed on the sagittal slices of the standard brain template (provided by the MRIcro package http://www.mricro.com), as illustrated in Figure 1-3 for each language test separately. Within the suprathreshold clusters, the neuroanatomical locations of peak voxels were summarized in Table 2 for each language test individually. 

**Table 1:** Demographic characteristics and neuropsychological performance.

% or Mean (SD)	Total (n=344)	70-79 years (n=205)	80-90 years (n=139)	p-value
Sex (% male)	45.6	47.3	43.2	0.45
Age	78.3 (4.8)	75.0 (2.4)	83.2 (2.7)	<0.001
Education (year)	11.8 (3.6)	11.9 (3.6)	11.5 (3.7)	0.77
COWAT	37.8 (12.3)	39.0 (12.2)	36.1 (12.4)	0.06
CF	16.0 (4.4)	16.7 (4.5)	14.8 (3.8)	<0.001
BNT	24.9 (3.4)	25.3 (3.2)	24.4 (3.6)	0.02

COWAT = Controlled Oral Word Association Task; CF = Category Fluency; BNT= Boston Naming Test.

Ratio of males was compared between the two age groups using the chi-square test. Age, years of education, and raw score on each of the three language tests were compared between two age groups using a univariate general linear model.

**Table 2:** Anatomical region and coordinates of peak voxels within the suprathreshold clusters correlated with three language tests in 344 participants aged 70-90 years.

Test	Cluster-level			Voxel-level				
	p (FWE)	size (n)		MNI coordinates			T value	Anatomical location (BA)
				X	Y	Z		
COWAT	0.002	466		-52	-38	-4	4.47	L posterior middle temporal gyrus (21)
	0.008	221		-20	-40	-38	4.46	L cerebellum
	0.035	275		60	-2	20	4.07	R precentral gyrus (6)
				56	14	24	3.88	R inferior frontal gyrus (44)
	0.008	257		30	-22	-10	3.28	R hippocampus
				32	-20	-12	3.27	R substantia nigra
	0.027	177		20	-42	-38	4.27	R cerebellum
CF	<0.001	3357		-32	-26	-10	5.14	L hippocampus
				-50	18	-12	4.38	L temporal pole (38)
				-42	0	2	3.67	L insula
				-38	24	-20	3.60	L orbitofrontal gyrus (47)
				-42	6	8	3.83	L inferior frontal gyrus (44)
	<0.001	847		-28	-62	-38	3.95	L cerebellum
BNT	0.001	1198		-24	4	-26	4.14	L parahippocampal gyrus (28)
				-34	6	-26	3.59	L temporal pole (38)
				-26	-10	-24	3.56	L hippocampus
	0.002	998		36	8	-28	4.38	R temporal pole (38)
				34	-20	-20	4.03	R parahippocampal gyrus (28)
				38	-16	-26	3.27	R fusiform gyrus (20)

The voxel-wise GM volumes were regressed on the three language test scores after controlling for age, years of education, sex, scanner, total intracranial volume (TIV), cardiovascular risk score, and handedness in the whole sample. The significance level was set at a voxel-level p<0.001 (uncorrected) combined with cluster-level p<0.05 (FWE-corrected) for the whole sample.

The cluster of voxels where GM volumes were positively correlated with COWAT were located in the left posterior middle temporal gyrus, right precentral and inferior frontal gyri, right hippocampus, right substantia nigra, and bilateral cerebellum (Figure 1). The positive GM correlates of CF were only located in the left hemisphere, including the hippocampus, parahippocampal gyrus, temporal pole, orbitofrontal gyrus, inferior frontal gyrus, insula, and cerebellum (Figure 2). The positive GM correlates of BNT were located in largely symmetrical positions of bilateral hemispheres, including the bilateral hippocampi, parahippocampal gyri and temporal poles, as well as the right fusiform gyrus (Figure 3). 

The conjunction analysis revealed no common GM correlates to all three language tests. However, there were common GM correlates (893 voxels) to CF and BNT in the left hippocampus and left parahippocampal gyrus (Figure S1A). The common GM correlates to COWAT and CF (34 voxels) were located in the left cerebellum (Figure S1B), and the common GM correlates to COWAT and BNT (8 voxels) were located in the right hippocampus (Figure S1C).

The correlations between structural laterality indices of ROIs and language tests in two age groups were shown in Table 3. We found a significantly positive correlation between CF and sLI of the inferior frontal gyrus (r=0.142, p=0.047) and an almost significant correlation between COWAT and sLI of the precentral gyrus (r=0.126, p=0.079) in the group of 70-79 years old, but both were not significant in the 80-90 year old group. Furthermore, Fisher's z test revealed a trend toward significant difference between the two age groups in the correlation between COWAT and sLI of the precentral gyrus (z=1.83, p=0.067), and the correlation between CF and sLI of the inferior frontal gyrus (z=1.65, p=0.099). After adjusted by Bonferroni correction for multiple comparisons, however, the differences between two age groups in the correlations between structural laterality indices of ROIs and language tests were not significant. The boundaries of bilateral ROIs were illustrated in Figure S2. Descriptive statistics of sLI for two age groups were presented in Table S1.

**Table 3:** Correlations between structural laterality indices of ROIs and language tests in two age groups.

		Young			Old				Difference	
		r	p-value		r	p-value			z	p-value
COWAT	PRE	0.126	0.079		-0.078	0.379			1.83	0.067
	IFG	0.102	0.154		0.094	0.289			0.07	0.94
		MTG	0.025	0.732		0.030	0.738		-0.04	0.968
CF	IFG	0.142	0.047*		-0.042	0.639			1.65	0.099
	TP	0.114	0.111		0.019	0.832			0.85	0.395
	HIPP	0.027	0.707		0.164	0.064			-1.23	0.219
BNT	TP	0.080	0.262		-0.025	0.777			0.94	0.347
	HIPP	-0.015	0.829		-0.012	0.896			-0.03	0.976
	FG	-0.021	0.772		-0.013	0.883			-0.07	0.944

The correlation coefficient (r) between structural laterality index of each ROI and each language test was calculated for each age group, using partial correlation model and controlling for age, years of education, sex, TIV, scanner, CVR, and handedness. Then the correlation coefficients were compared between the two age groups, using a Fisher’s z-transformation of the r values and the level of significance was determined.

Abbreviation for ROIs: PRE = precentral gyrus; IFG = inferior frontal gyrus (including opercular part and triangular part); MTG = middle temporal gyrus; TP = superior temporal pole; HIPP = combined hippocampus and parahippocampal gyrus; FG = fusiform gyrus.

* p<0.05

In addition, we divided the whole sample into two groups according to the two scanners, and then performed correlation analysis between voxel-wise GM volumes and language tests for each scanner group. We found similar patterns in the GM correlates of language tests between the two scanner groups (see Figure S3). Furthermore, we performed correlation analyses between structural laterality indices and language tests for each scanner group. The correlation coefficients were not significantly different between the two groups. 

## Discussion

Our study demonstrated that three language tests were all positively correlated with GM volumes in the frontal, temporal and parietal lobes in the non-demented elderly adults. Our results also displayed distinct GM correlation patterns for each language test. Although all three tests involve word retrieval and articulation, they differ in the strategies applied in word searching, selection and inhibition processes. Both verbal fluency tests, COWAT and CF, measure the efficiency of word generation, but differ in how the output is induced by either phonemic or semantic cues. The confrontational naming test, BNT, does not require automatic word generation, but evaluates the naming ability induced by visual stimulus. The GM correlation patterns of three language tests were discussed individually, and the common and specific features were identified. 

### Grey Matter Correlates of COWAT

As a classical phonemic fluency test, GM correlates of COWAT were mainly located in the frontal, temporal and subcortical areas in the non-demented adults aged 70-90 years. The relationship between COWAT and the frontal GM volumes, in particular the precentral and inferior frontal gyri, has been consistently demonstrated in functional neuroimaging studies, which suggest the frontal regions are involved in executive control and search strategies [60-62]. Our results were also consistent with previous structural MRI studies, which showed the patients with frontal lobe lesions performed poorly on phonemic fluency tests [63], Moreover, we found that COWAT was positively correlated with the left posterior middle temporal GM volumes. Prior studies have shown that this brain area, along with the inferior frontal gyrus, is involved in executive control of demanding language tasks [13,64-66]. A positive correlation between the substantia nigra and COWAT was also observed in this study, consistent with the involvement of this region in planning and execution of articulatory movement that is heavily engaged in this phonemic fluency test [67,68]. We noted that the frontal GM correlates of COWAT was only located in the right hemisphere, instead of a left-hemispheric dominance that is often found in healthy younger adults [28]. This observation might reflect the reduction of hemispheric asymmetry in late life, consistent with the findings of prior fMRI studies on the neural substrates of language tasks in older adults [69]. 

### Grey Matter Correlates of CF

Our results showed that this semantic fluency test was correlated with GM volumes in the left hemisphere, including the frontal and temporal lobes, as well as the hippocampus, insula and cerebellum. Similar to COWAT, CF had the GM correlates in the frontal lobe, which may also indicate the involvement of this region in executive control and search strategies that are essential to both verbal fluency tests [60-62]. However, the extent and exact locations of the frontal GM correlates of two verbal fluency tests were different, with a bigger cluster of voxels in the precentral and inferior frontal gyrus for COWAT, and a smaller cluster of voxels in the orbitofrontal and inferior frontal gyrus for CF. This difference is coherent with previous studies, which suggests that phonemic fluency tasks have greater executive demands and require more frontal activation than semantic fluency tasks, and the frontal neural basis is located in more dorsal positions than that of semantic fluency tasks [70-72]. Our study also showed that the positive GM correlates of CF were located in the temporal and hippocampal regions. Although the involvement of the hippocampus and its neighbouring areas in episodic memory has been well-established [73-75], their roles in semantic processes is still a topic of debate [76-79]. In a recent fMRI study Ryan and colleagues found the hippocampal activation during performing both semantic and episodic retrieval tasks in healthy adults [80]. Previous studies also showed that the patients with the temporal atrophy such as Alzheimer’s disease often had semantic impairments [81,82]. Our findings provide further support for the roles of the hippocampus and its adjacent brain regions in the retrieval of semantic knowledge. This study also showed that the insula was correlated with CF. A previous study revealed that the left insula atrophy was correlated with verbal generation difficulty in healthy participants across 19-88 years [83]. Our findings are consistent with the role of the insula in articulation planning [84-86]. 

### Grey Matter Correlates of BNT

The present study showed a symmetrical pattern in the GM correlates of BNT, which were located in the bilateral hippocampi, parahippocampal gyri and temporal poles. In contrast to the two verbal fluency tests, BNT had no significant GM correlates in the frontal lobe. Evidence from lesion studies revealed that patients suffering from frontal lobe damage showed no deficit in naming ability, but severe impairment in verbal fluency [87,88]. The evidence may suggest that the naming test has a relatively small demand on executive function and attention, different from verbal fluency tests [89]. As shown in the conjunction analysis, BNT and CF were both correlated with GM volumes in the left hippocampus and left parahippocampal gyrus, consistent with the involvement of these regions in semantic retrieval [80], an essential cognitive component for both tests [90]. The temporal pole was also found to be correlated with BNT in this study, which is possibly concordant with the role of this region as a hub area to converge different sources of information about an object, in order to assist semantic retrieval for the naming task [91-93]. The correlation of BNT with the fusiform gyrus may indicate the involvement of this region in processing visual information regarding an object, as revealed in previous studies [28,94]. Moreover, we found that three language tests all had GM correlates in the cerebellum. Although only a few studies investigated the role of cerebellum in language, they have shown that the cerebellum has a contribution to speech production and verbal working memory [84,95,96], which are important components for the three language tests. 

### Language Lateralization in Late Life

Our study showed that positive GM correlates of language tests were located in the right frontal lobe for COWAT and the bilateral temporal lobes for BNT. This finding indicated a less leftward GM correlation pattern for language tests, consistent with the reduction of hemispheric asymmetry in older adults as observed in previous fMRI studies [37-39]. The trend of changing language lateralization with age has been demonstrated by a recent fMRI study, which shows the left-hemispheric dominance increasing with age between 5-20 years, reaching a plateau at 20-25 years, and then slowly declining afterwards [43]. Different from fMRI studies that directly compare bilateral neural activities related to a language task, structural MRI studies have used structural laterality to investigate language lateralization. Evidence has shown that language lateralization is associated with structural laterality; moreover, the degree of leftward structural asymmetry is positively correlated with language performance, consistent with the notion of language lateralization [31,34-36]. Using structural asymmetry indices that were computed with regional GM volumes, we explored the relationship between structural asymmetry indices and language performance in two age groups (70-79 and 80-90 years old). We noted that the difference between two groups was approaching significance (p<0.1), with stronger correlations between leftward asymmetry of the frontal regions and two language tests (CF and COWAT) in the younger group than those in the older group. The results, together with our findings of less leftward patterns in voxel-wise GM correlations of language tests, suggest a further declining of language lateralization in different stages of late life. 

Our study is subject to several limitations. Firstly, as the ageing brain often undergoes regional structural changes, such as GM atrophy and WM disruption, the reliability of brain registration and segmentation could be affected in processing brain images of the elderly adults [97]. However, we used an advanced DARTEL method, which has shown a better registration effect compared to other methods [98], to improve the accuracy of image processing. Secondly, the change of scanner in the middle of the study is a potential limitation though the two scanners were made by the same manufacturer and used the same parameter settings. The validation tests showed that the two scanner groups had similar neuroanatomical correlation patterns with language tests, and the correlations between structural laterality indices and language tests did not show any significantly difference between the two scanner groups. Moreover, the inclusion of scanner type as a covariate could minimize the likelihood of its influence on the relationship between voxel-wise GM volumes and language tests. 

In conclusion, our study demonstrated distinctively positive correlation patterns between voxel-wise GM volumes and three standardized language tests (COWAT, CF and BNT) in a large sample of non-demented, community-dwelling adults aged 70-90 years. Our results showed that COWAT was mainly correlated with the right frontal and left temporal GM volumes, CF with the left frontal and left temporal GM volumes, and BNT with bilateral temporal GM volumes. The neuroanatomical locations of these GM correlates were largely consistent with the findings of fMRI studies on neural substrates of language tasks, and they also indicated a reduction of hemispheric asymmetry as shown in the pattern of GM correlates of language tests. In addition, we found a trend toward significant difference in the correlations between structural laterality index and language tests between two age groups (70-79 and 80-90 years old), with stronger correlations in the younger age group than those in the older age group. This difference may suggest a further decline of language lateralization with age in late life.

## Supporting Information

Figure S1 **Common GM correlates of language tests.** The conjunction analysis showed the common voxels where GM volumes are positively correlated with different language tests in 344 participants aged 70-90 years. These common GM correlates in colour red were superimposed on the sagittal slices of the brain template. A) common GM correlates to CF and BNT; B) common GM correlates to COWAT and CF; C) common GM correlates to COWAT and BNT.
(TIF)

Figure S2 **Bilateral ROIs for each language test.** Based on the locations where voxel-wise volumes were positively correlated with three language tests in the whole sample, region-of-interests (ROIs) for each language test were determined. The boundary of each ROI was delineated using the Automated Anatomical Labelling atlas (AAL), and demonstrated by superimposing on the sagittal slices of the brain template. The slices were at 4 mm intervals between and including -80 mm and 76 mm. A) bilateral ROIs of COWAT; B) bilateral ROIs of CF; C) bilateral ROIs of BNT.
(TIF)

Figure S3 **Grey matter correlates of three language tests in two scanner groups.** Brain regions where voxel-based GM volumes were positively correlated with three language tests in two scanner groups were superimposed on the 3D brain templates. The figures shown in the 1^st^ column were for the group of Scanner 1, while the figures in the 2^nd^ column were for the group of Scanner 2. The figures for each language test were demonstrated in three rows. A) Grey matter correlates of COWAT; B) Grey matter correlates of CF; C) Grey matter correlates of BNT.
(TIF)

Table S1 **Descriptive statistics of structural laterality indices (sLI) of region-of-interests (ROIs) in two age groups.**
(DOCX)
