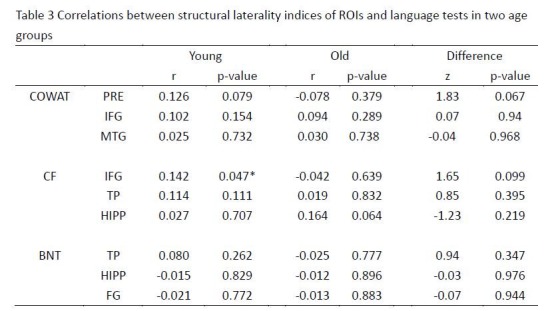# Correction: Grey Matter Correlates of Three Language Tests in Non-demented Older Adults

**DOI:** 10.1371/annotation/2e4d150f-c396-4867-b170-e43ccff9fcd7

**Published:** 2014-01-17

**Authors:** Haobo Zhang, Perminder S. Sachdev, Wei Wen, Nicole A. Kochan, John D. Crawford, Henry Brodaty, Melissa J. Slavin, Simone Reppermund, Kristan Kang, Julian N. Trollor

During production formatting errors were introduced into Table 3, "Correlations between structural laterality indices of ROIs and language tests in two age groups." Every number in the fifth row has been incorrectly moved one cell to the right. The correct version of Table 3 can be found here: 

**Figure:**