# Supplementary figures and images for: Grey Matter Correlates of Three Language Tests in Non-demented Older Adults
Source: PLoS One. 2013 Nov 5;8(11):e80215. doi: 10.1371/journal.pone.0080215 (PMC3818244; doi:10.1371/journal.pone.0080215)

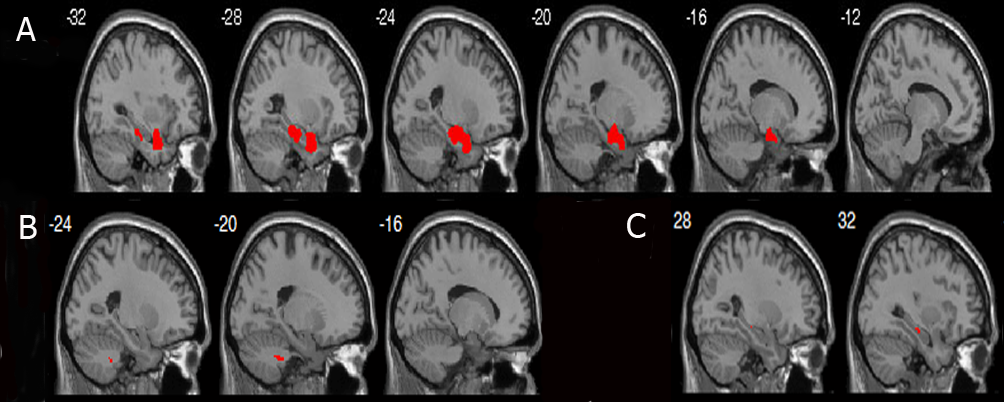

Supplement: Figure S1 — Common GM correlates of language tests. The conjunction analysis showed the common voxels where GM volumes are positively correlated with different language tests in 344 participants aged 70-90 years. These common GM correlates in colour red were superimposed on the sagittal slices of the brain template. A) common GM correlates to CF and BNT; B) common GM correlates to COWAT and CF; C) common GM correlates to COWAT and BNT. (TIF) [file pone.0080215.s001.tif]

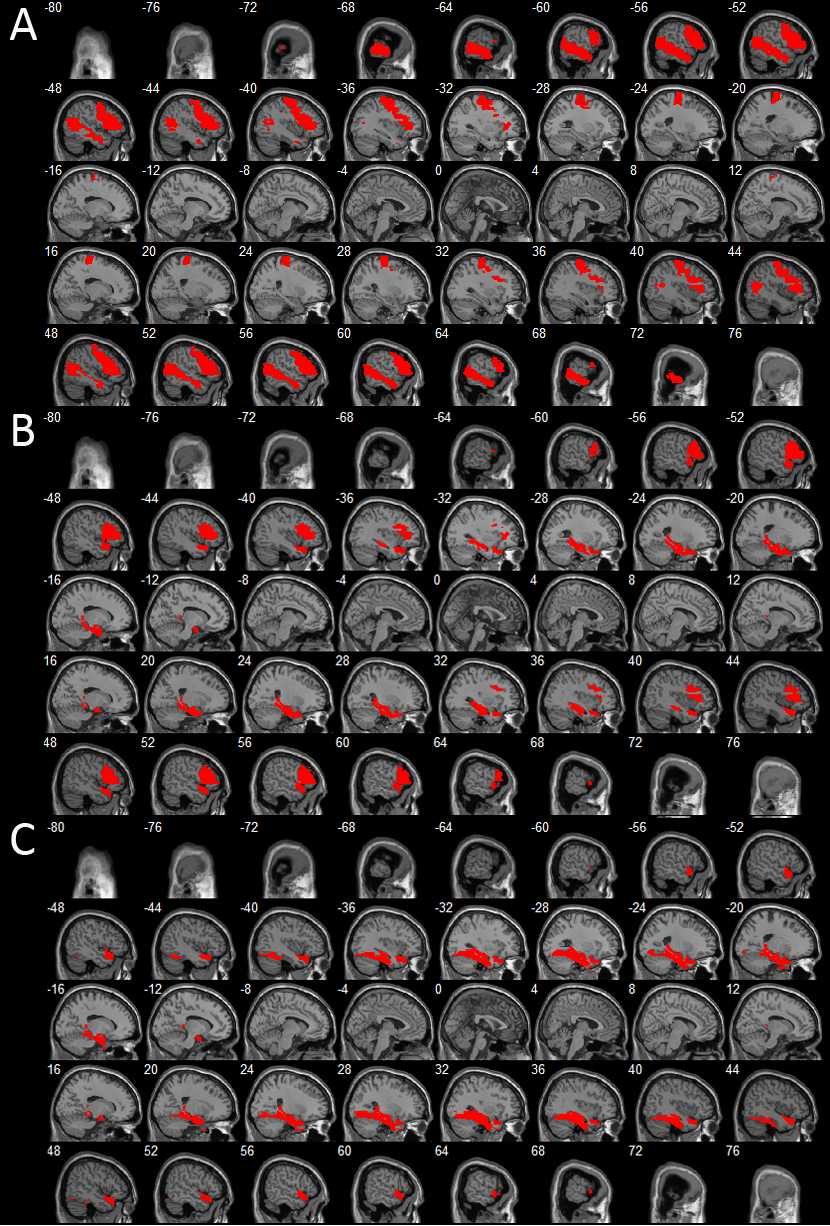

Supplement: Figure S2 — Bilateral ROIs for each language test. Based on the locations where voxel-wise volumes were positively correlated with three language tests in the whole sample, region-of-interests (ROIs) for each language test were determined. The boundary of each ROI was delineated using the Automated Anatomical Labelling atlas (AAL), and demonstrated by superimposing on the sagittal slices of the brain template. The slices were at 4 mm intervals between and including -80 mm and 76 mm. A) bilateral ROIs of COWAT; B) bilateral ROIs of CF; C) bilateral ROIs of BNT. (TIF) [file pone.0080215.s002.tif]

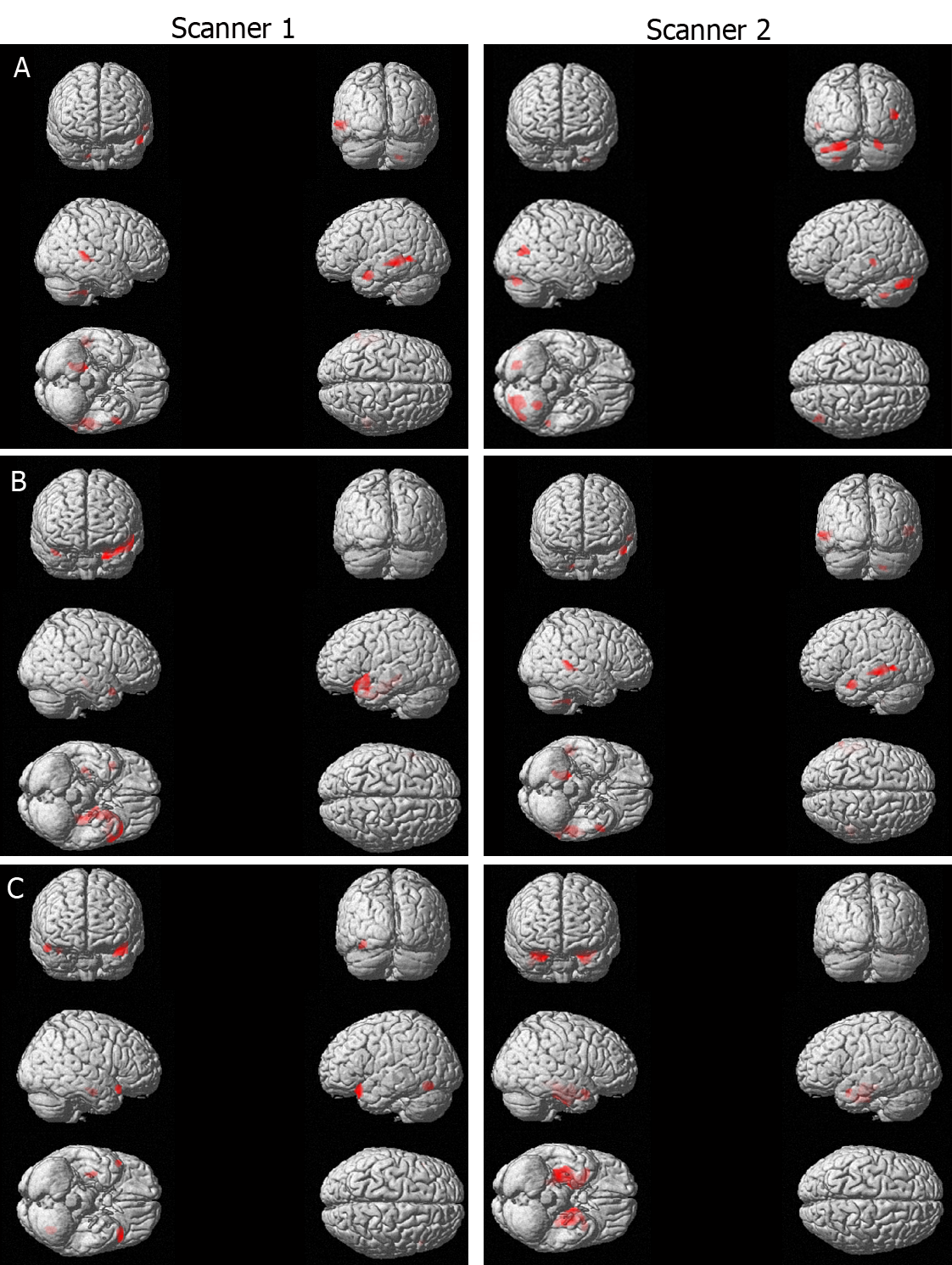

Supplement: Figure S3 — Grey matter correlates of three language tests in two scanner groups. Brain regions where voxel-based GM volumes were positively correlated with three language tests in two scanner groups were superimposed on the 3D brain templates. The figures shown in the 1st column were for the group of Scanner 1, while the figures in the 2nd column were for the group of Scanner 2. The figures for each language test were demonstrated in three rows. A) Grey matter correlates of COWAT; B) Grey matter correlates of CF; C) Grey matter correlates of BNT. (TIF) [file pone.0080215.s003.tif]
