# Supplementary material for: Grey Matter Correlates of Three Language Tests in Non-demented Older Adults
Source: PLoS One. 2013 Nov 5;8(11):e80215. doi: 10.1371/journal.pone.0080215 (PMC3818244; doi:10.1371/journal.pone.0080215)
Supplement: Table S1 — Descriptive statistics of structural laterality indices (sLI) of region-of-interests (ROIs) in two age groups. (DOCX) [file pone.0080215.s004.docx]

Supplementary Table S1.

Descriptive statistics of structural laterality indices (sLI) of region-of-interests (ROIs) in two age groups

|  | Young  Mean (SE) | Old  Mean (SE) | p-value |
| --- | --- | --- | --- |
| PRE | 0.026 (0.003) | 0.022 (0.003) | 0.27 |
| IFG | 0.011 (0.003) | 0.011 (0.003) | 0.85 |
| MTG | 0.048 (0.003) | 0.048 (0.003) | 0.99 |
| TP | -0.017 (0.003) | -0.022 (0.003) | 0.23 |
| HIPP | 0.001 (0.002) | -0.002 (0.002) | 0.29 |
| FG | 0.005 (0.002) | 0.000 (0.003) | 0.18 |

For each region-of-interest (ROI), the bilateral GM volumes (V_left_ and V_right_) were calculated as the sum of voxel-wise GM volumes within each hemispheric ROI. The structural laterality index (sLI) of each ROI was computed individually with the formula sLI = (V_left_ - V_right_)/(V_left_ + V_right_). The comparison of sLI between two age groups (70-79 vs 80-90 years old) was performed after controlling for scanner, sex and TIV. Descriptive statistics of sLI in two age groups, including mean and SE values, were provided.

Abbreviation for ROIs: PRE = precentral gyrus; IFG = inferior frontal gyrus (including opercular part and triangular part); MTG = middle temporal gyrus; TP = superior temporal pole; HIPP = combined hippocampus and parahippocampal gyrus; FG = fusiform gyrus.
